# Mitral Valve Repair in Young Rheumatic Patients

**DOI:** 10.5935/abc.20190215

**Published:** 2019-10

**Authors:** Pablo Maria Alberto Pomerantzeff

**Affiliations:** Faculdade de Medicina da Universidade de São Paulo, São Paulo, SP - Brazil; Unidade Cirúrgica de Cardiopatia Valvar - Instituto do Coração do Hospital das Clínicas da Faculdade de Medicina da Universidade de São Paulo, São Paulo, SP - Brazil

**Keywords:** Heart Defects Congenital, mitral Valve Insufficiency/surgery, hypertension, Pulmonary, Reoperation, Cardiopathy, Rheumatic

Mitral valve repair surgery presents excellent immediate and late results in young patients with valve disease resulting from chronic rheumatic heart disease such as those reported in the article by Cruz et al.^1^ in this issue.

There is agreement in the literature on the lower morbidity and mortality of patients submitted to mitral repair surgery in relation to valve replacement, but there is no uniformity of results of mitral valve reconstruction in patients with lesions resulting from rheumatic fever, perhaps, due to the recurrence of new rheumatic episodes in the evolution of these patients.^2^

In the work of Cruz et al.,^1^ they found that the time of use of vasoactive drug is an independent predictor for unfavorable outcomes in the immediate and late postoperative, while residual mitral valve insufficiency was associated with reoperation and both tricuspid insufficiency and pulmonary hypertension were associated with unfavorable outcomes. We should consider some points. Mitral valve insufficiency surgery in rheumatic patients should be performed from the anatomic point of view when there is still a pliable anterior leaflet, at the appropriate time, neither early nor late, so that the surgery is performed before the development of pulmonary hypertension and tricuspid insufficiency, although it is known that many patients only seek care already in advanced stages of the disease.

As reported in our study,^2^ valvar reconstruction requires the surgeon a perfect knowledge of anatomy and the multiplicity of existing techniques. In addition, an evaluation of the leaflets, a mitral valve annulus, chordae tendineae and papillary muscles should be performed systematically during the surgery. Surgery should be performed with Doppler echocardiographic examination through the transesophageal tube.

When the anterior leaflet is thickened and there is a significant mitral insufficiency, it is technically possible to correct the reflux by means of reconstructive techniques, but this valve will most likely become stenotic due to the restriction of movement caused by this thickening of the anterior leaflet of the mitral valve, impairing its opening.

Other anatomical details are important. The normal mitral valve presents a zone of coaptation between the anterior and posterior leaflets, around 6 to 8 mm, coaptation that we should pursue in the performance of a mitral valve repair surgery because this adequate coaptation will favor a good long-term evolution. This coaptation is easily proven in the intraoperative period, with the test of saline solution with the filling of the left ventricle, making the mitral valve competent after the repair surgery, and the use of brushing with methylene blue in the atrial line of coaptation. Besides this good coaptation, a successful mitral valve repair must have an adequate valve area after the procedure, without ever causing stenosis.

We must not forget that myocardial protection in cardiac surgery is fundamental. If this protection is performed properly and we get good mitral valve reconstruction, confirmed by echocardiography in a patient with good preoperative ventricular function, the need for vasoactive drugs in the immediate postoperative period will certainly below.

Several studies^3^ have shown a direct relationship with good results, that is, the durability of the repair and the volume of mitral repairs made by a given service.

Undoubtedly, the cardiovascular surgeon who is interested in mitral valve reconstruction should accompany for some time an institution that routinely uses this technique.
